# Exploring the experience of boarded psychiatric patients in adult emergency departments

**DOI:** 10.1186/s12888-021-03446-1

**Published:** 2021-09-27

**Authors:** Daniel Major, Katherine Rittenbach, Frank MacMaster, Hina Walia, Stephanie D. VandenBerg

**Affiliations:** 1 Present Address: Faculty of Science and Technology, Mount Royal University, Calgary, AB Canada; 2 Addictions & Mental Health Strategic Clinical Network, Alberta Health Services, Calgary, AB Canada; 3 Department of Psychiatry, Alberta Health Services, Calgary, AB Canada; 4 Department of Emergency Medicine, Alberta Health Services, C231 Foothills Medical Centre, 1403 29 St NW, Calgary, AB T2N 2T9 Canada; 5 Cumming School of Medicine, University of Calgary, Calgary, AB Canada

**Keywords:** Boarding, Emergency department, Mental health care, Safety

## Abstract

**Background:**

This study quantifies the frequency of adverse events (AEs) experienced by psychiatric patients while boarded in the emergency department (ED) and describes those events over a broad range of categories.

**Methods:**

A retrospective chart review (RCR) of adult psychiatric patients aged 18–55 presenting to one of four Calgary EDs (Foothills Medical Centre (FMC), the Peter Lougheed Centre (PLC), the Rockyview General Hospital (RGH), and South Health Campus (SHC)) who were subsequently admitted to an inpatient psychiatric unit between January 1, 2019 and May 15, 2019 were eligible for review. A test of association was used to determine the odds of an independent variable being associated with an adverse event.

**Results:**

During the study time period, 1862 adult patients were admitted from EDs (city wide) to the psychiatry service. Of the 200 charts reviewed, the average boarding time was 23.5 h with an average total ED length of stay of 31 h for all presentations within the sample. Those who experienced an AE while boarded in the ED had a significantly prolonged average boarding time (35 h) compared to those who did not experience one (6.5 h) (*p* = 0.005).

**Conclusions:**

The length of time a patient is in the emergency department and the length of time a patient is boarded after admission significantly increases the odds that the patient will experience an AE while in the ED. Other significant factors associated with AEs include the type of admission and the hospital the patient was admitted from.

## Background

Adult Emergency Departments (EDs) in Calgary are facing a crisis in boarding patients admitted to psychiatric in-patient units. In psychiatric emergency care, “boarding” describes the holding of patients in the ED after the decision to admit has been made by a staff psychiatrist and a bed request has been submitted [1]. Psychiatric patient boarding in the ED is particularly suboptimal due to the lack of therapeutic resources and environments available, delaying the initiation of treatment and causing downstream impacts for ED wait times and bottlenecks for other emergency services i.e. police and emergency medical services (EMS). Despite this, psychiatric inpatients face exorbitantly higher boarding times than any other service in the hospital [2–5]. In the US, 12.5% of all ED visits involved a mental health or substance abuse diagnosis, and 41% of patients presenting with a mental health or substance abuse issue were admitted [6].

Currently there is substantial literature examining the boarding of psychiatric patients in the EDs of acute care facilities in North America, however, this body of literature appears to lack the consideration and exploration of unanticipated events associated with boarding adult patients. Adverse Events (AEs) in the emergency department have been defined as unintended harms resulting from the delivery of care [7]. The Canadian Patient Safety Institute acknowledges there are special considerations to be made for safety issues surrounding mental health patients, including: suicide and self-harm, violence and aggressive behavior, restraint use and seclusion, and absconding [8]. In this study, adverse events from a psychiatric emergency perspective could include: the need to initiate mechanical and/ or chemical restraints after admission and while still in the ED; the application of verbal interventions and/ or physical restraint by staff members including security personnel; attempts to self-harm; verbal and physical assault on staff; the restriction of movement by the patient due to increasing agitation (i.e. locking the door to the patient’s room); elopement attempts; increasing levels of agitation; leaving against medical advice; the patient’s admission condition being changed to high observation due to behavior while in Emergency after admission to a regular bed has already been deemed appropriate; and/or the need for certification under the Mental Health Act after admission has already been deemed appropriate.

This study aims to quantify the frequency and characteristics of adverse events (AEs) experienced by psychiatric patients who are boarded and test for associations related to severity of illness. We hypothesize, based on review of North American literature, that the incidence of AEs per patient record will approach 50% and will most often involve increasing levels of agitation, the use of verbal interventions and/or mechanical/chemical restraints and/or the use of security personnel involvement to intervene in the care of the boarded psychiatric patient.

## Methods

### Study design and time period

A retrospective chart review (RCR) of adult psychiatric patients aged 18–55 presenting to one of four Calgary EDs (Foothills Medical Centre (FMC), the Peter Lougheed Centre (PLC), the Rockyview General Hospital (RGH), and South Health Campus (SHC)) who were subsequently admitted to an inpatient psychiatric unit between January 1, 2019 and May 15, 2019 were eligible for review. Patients admitted to non-psychiatric services first or transferred to another hospital site were excluded. Ethics approval was obtained from the University of Calgary Research Ethics Board. A clinical query on an electronic medical record software (Sunrise Clinical Manager) using “mental health nursing transfer report” or “admission to psychiatric services” or “admission to mental health unit” was used to determine the pool of participants. Using this pool, a single unblinded researcher randomly selected 50 charts from each of the four adult acute care centers. The chart review included: the record of treatment, multidisciplinary notes and nursing notes from the physical charts; clinical entries from emergency nurses and psychiatric emergency assessment staff on Sunrise Client Manager (SCM) (ED reporting system); and electronic reports on Perspective (Protective Services (security) reporting system).

### Study setting

Calgary is a major urban center in western Canada, with a population of 1,669,272 as at 2018–19 [9]. Emergency care is provided to adult patients in Calgary through four Emergency Departments and two urgent care centres. In 2018–19, there were 476,013 presentations to emergency departments and 185,455 presentations to urgent care in Calgary [9]. All emergency departments are staffed by full-time emergency physicians and psychiatric assessment services are available for each department. Calgary has 287 acute care adult psychiatric beds spread across the four adult hospitals. Inpatient psychiatric beds vary by site and acuity and range from High observation beds, akin to a psychiatric intensive care bed, to short stay (transitioned to community within 72 h).

### Population

All adult patients between 18 and 55 years of age with an admission to a psychiatric unit after a presentation to a Calgary ED were included. To eliminate confounding factors, patients who were discharged from the ED but had an admission report completed or transferred to another site for admission were excluded from potential chart review.

### Outcome measures

The primary outcome measure was the type and frequency of AEs experienced by psychiatric patients who were boarded based on the following a priori variables: gender, length of stay, time spent boarded, admission type, living situation, Canadian Triage and Acuity Scale (CTAS) score, time of admission, day of admission and arrival method. Secondary outcomes of interest included: the quantification and description of discrete AE types; average length of boarding time experienced by a psychiatric inpatient; type of chemical restraint used; frequency that security personnel are required for intervention; boarding times experienced based on day of admission and time of admission; boarding times experienced based on living situation and the frequency of admission certification under the Mental Health Act (Table 2).

### Data analysis

Demographic data and individual AEs are reported in frequencies and analyzed as categorical variables using chi-square for strength of association. Length of stay and boarding time are reported as a continuous variable and analyzed using analysis of variance (ANOVA). The independent variables associated with both the length of boarded time and an AE were analyzed and presented as a function of a priori variables which included age, gender, hospital, type of admission and other patient and emergency department demographics.

## Results

**Fig. 1:**
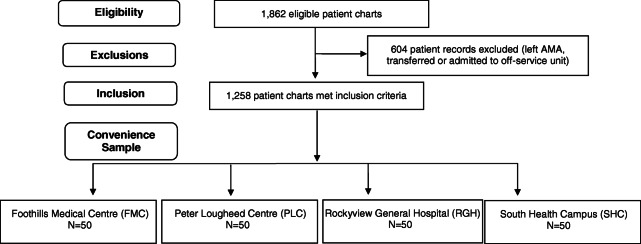
Flow diagram of study profile

During the study period, a total of 1862 adult patient records were potentially eligible for inclusion. Of these, 1258 patient records were included for potential chart review (98% admitted, 2% left against medical advice). 200 charts were identified as a random sample, with 50 charts from each of the four adult emergency departments (Fig. 1). The average age of participants was 34 with just over half of the sample identifying as a man and just over 10% presenting with no fixed address. Average emergency department length of stay (ED LOS) was 31.1 h, with an average length of boarding of 23.6 h (Table 1). The three most common admission diagnoses were: severe depressive episode (28, 14%), nonorganic psychosis (28, 14%), and adjustment disorder (24, 12%). Of the patients who presented at triage, just over half were assigned Canadian Triage Acuity Scale (CTAS) 2. Roughly half the admission orders were entered between 15:01–23:00. Most admissions occurred on Wednesday (21%) and the fewest on Thursday (8%). Most patients arrived by walk-in (55.5%), however, 20% arrived with police, an additional 20% arrived with EMS, and the remainder were either placed under a Form 1 of the Alberta Mental Health Act on site, or arrived at triage with both EMS and police. In addition, 119 (59.5%) patients were placed under a second Form 1 (admission certificate) by a psychiatrist.

**Table 1:** Demographic Information of Patients admitted to an acute care psychiatric bed from dour adult Emergency Departments in Calgary

	Mean (Range)
Age (years)	34 (18–55)
ED LOS (minutes)	1868 (213–16,091)
Time Boarded (minutes)	1415 (22–15,856)
	**Frequency (relative)**
Gender	
Woman	95 (47.5%)
Man	105 (52.5%)
Admission type	
High Observation/High Acuity	29 (14.5%)
Hospitalist	1 (0.5%)
Normal	108 (54%)
Short stay	58 (29%)
Short stay with high observation/high acuity	3 (1.5%)
None	1 (0.5%)
Living Situation	
Housed	178 (89%)
No Fixed Address	22 (11%)
CTAS Score	
1	4 (2%)
2	111 (55%)
3	64 (32%)
4	19 (9.5%)
5	2 (1%)
Time of Admission	
07:00–15:00	51 (25%)
15:01–23:00	96 (48%)
23:01–07:00	53 (26.5%)
Admission Day	
Monday	33 (16.5%)
Tuesday	28 (14%)
Wednesday	42 (21%)
Thursday	16 (8%)
Friday	27 (13.5%)
Saturday	30 (15%)
Sunday	24 (12%)
Arrival Method*	
Police	40 (20%)
EMS	38 (19%)
EMS + Police	6 (3%)
F1 on site	5 (2.5%)
Walk-in	111 (55.5%)

The ED LOS, boarding time, admission type, and hospital of presentation were each significantly associated with at least one AE (*p* < 0.05). For admission type, patients had 82% higher odds of experiencing an AE if admission was to a high observation (high acuity) versus a normal bed admission (95% CI: (0.05, 0.64); *p*-value = 0.008); and 92% higher odds if admission was to a high observation (high acuity) bed versus short stay (95% CI: (0.02, 0.82); p-value = 0.0001).

116 (58%) patients experienced at least one AE while boarded. The five most frequent AE categories experienced by patients were: increasing agitation (38%), benzodiazepine use (34%), sleep problems (21.5%), non-security verbal intervention (20.5%), and Protective Services physical and/ or verbal intervention (14%) (Table 2).

**Table 2:** Documented frequency of patients experiencing one or more AEs by category and admitted to psychiatry service while being boarded in emergency departments in Calgary

	Frequency of AE during boarding
Mechanical restraint application	1 (0.5%)
Environmental restraint application (i.e. locked door)	24 (12%)
Chemical restraint (CR) application	28 (14%)
Protective Services standby during CR	13 (6.5%)
Protective Services physical restraint during CR	8 (4%)
Chemical sedation (e.g. for anxiety)	55 (27.5%)
Types of medications administered*	
Benzodiazepine	68 (34%)
Ativan	62 (31%)
Diazepam	3 (1.5%)
Clonazepam	3 (1.5%)
Quetiapine	14 (7%)
Haloperidol	18 (9%)
Olanzapine	11 (5.5%)
Risperidone	5 (2.5%)
Loxapine	1 (0.5%)
Zuclopenthixol	2 (1%)
Chlordiazepoxide	0 (0%)
Haldol+Ativan intramuscular	7 (3.5%)
Protective Services personnel intervention	33 (16.5%)
Verbal intervention	25 (12.5%)
Physical restraint (no chemical restraint)	8 (4%)
Non-security personnel verbal intervention	41 (20.5%)
Clinical staff verbally assaulted by patient	8 (4%)
Clinical staff physically assaulted by patient	4 (2%)
Elopement attempted one or more times	7 (3.5%)
Self-harm or self-harm attempt one or more times	1 (0.5%)
Increasing agitation, anxiety, or severity of behavior	76 (38%)
Removal of personal belongings or comfort items	21 (10.5%)
Sleep problems	43 (21.5%)
Inappropriate use of substances in ED room	3 (1.5%)
First Formal Certification (Form 1) in ED	24 (12%)
Left AMA	4 (2%)

## Discussion

### Interpretation of findings

This is the first study, to our knowledge, that not only quantifies but explores the character of the type of adverse events that psychiatric patients experience while boarded in the ED. It should be noted that the study definition of AE included administration of medications that may be interpreted as part of routine patient care or as beneficial, as opposed to harmful, to the patient, depending on the setting they are administered in. For example, the unscheduled use of benzodiazepines may be a routine part of psychiatric patient care or requested by the patient for anxiolysis (we excluded cases where benzodiazepines were required for alcohol withdrawal management). This could be considered an unplanned event however we chose to classify this as an adverse event to reflect the need for treatment of agitation to maintain patient and staff safety, in keeping with recommendations made by working groups such as the American Association for Emergency Psychiatric Project [10]. We classified other variables as adverse events to reflect the disruption a patient experiences with respect to sleep, diet and social interaction while held in the emergency department.

Psychiatric patients appear to experience longer boarding times than those patients being admitted to non-psychiatric services [2–5]. Examining AEs experienced by medical non-psychiatric patients while boarding has been undertaken by Liu et al. [2]. One study examined predictors of prolonged ED visits by adult patients ≥65 yo, which included the use of chemical or physical restraints as a variable with a positive correlation to prolonged stay [11]. Of note, there is a remarkable lack of studies focused on the psychiatric emergency patient population in Canada.

Our study found that the patients requiring a higher threshold of care are waiting longer and are more likely to have an AE. Patients requiring a high observation bed boarded for 78 h compared to the zone average boarding time of 24 h. As reviewed in Zeller et al., literature out of the US presents a range of boarding times between 2.5 and 34 h for adult psychiatric patients [4]. AEs were significantly associated with ED LOS and boarding time. While this could be attributed to the severity of their condition, this may also be attributed in part to the amount of time they are spending in the ED.

Our results do not describe the total number of AEs experienced by the sample population during the study time frame, rather, the occurrence of an AE was a discrete variable indicating whether this type of event happened one or more times during boarding. It is therefore an indicator of how many patients experienced this type of AE, but it does not capture the frequency with which these AEs occur. Future studies should examine the frequency of AEs as a variable of consideration.

Lastly, patients were admitted with a spectrum of psychiatric diagnoses which makes it difficult to develop operational protocols or guidelines for specific psychiatric disease entities in order to reduce boarding times for common psychiatric presenting concerns.

### Strengths and limitations

This study is the first to provide a detailed look at the experience of acutely ill psychiatric patients awaiting movement out of the emergency department and into an inpatient area. It is also the first to incorporate information from the Protective Services reporting system which presents a rich data set describing interactions with this key stakeholder in the emergency department experience for these patients. It is limited by the small sample size as well as the fact that the sample may be biased to more acutely ill patients as we excluded patients who were transferred between sites. We also must accept that our study is limited by inevitably imperfect documentation practices, as we rely on detailed reporting to capture our data. Finally, even though the study looked at one geographical area, each adult emergency department has access to different physical layouts of the psychiatric emergency services as well as access to different psychiatric emergency staff which introduces heterogeneity to the independent variables based on site.

### Clinical implications

The importance of understanding the reality of the conditions that psychiatric ED patients face while waiting for in-patient placement cannot be overstated. In 2018–19, the average LOS for admitted patients in emergency departments in Calgary was 9.75 h [12]. The admitted patients in our study spent, on average, 31.1 h in the emergency department. This sample represents the large disparity in wait times experienced by psychiatry patients who are admitted to acute care in-patient units. Our results are in line with other literature published in North America which indicates higher average ED LOS for psychiatric in-patients.

This study is important to emergency medicine as it allows for a deeper understanding of the patient experience in the emergency department and may identify areas that require advocacy and relationship building between ED staff and psychiatry colleagues. It is also imperative to maintain patient safety while reducing workplace violence towards staff, specifically in the emergency department [10].

### Research implications

The evaluation of the model of service delivery for psychiatric emergency department patients is an area for future exploration. This could include exploring a city-wide pilot study for a dedicated psychiatric emergency department as a service delivery model or the use of crisis stabilization units (CSUs) [4].

## Conclusions

Our study provides unique insight into the unplanned and unpredictable events commonly experienced by acutely ill psychiatric patients in the emergency department while awaiting their inpatient bed. As hypothesized, over 50% of charts reviewed documented at least one adverse event that directly affected the patient. The total time spent in the emergency department and while boarded as well as the type of admission unit requested and the distinct emergency department a patient received care from significantly affected the number of AEs experienced by the patient. These findings may be used to further compare inpatient experiences of AEs as well as to advocate for appropriate psychiatric care for patients while in the emergency department.

## Data Availability

Upon request from the principle author.

## Abbreviations

AE: Adverse Event
ANOVA: Analysis of Variance
CI: Confidence interval
CSU: Crisis Stabilization Unit
CTAS: Canadian Triage Acuity Scale
ED: Emergency Department
EMS: Emergency Medical Services
LOS: Length of Stay
RCR: Retrospective chart review
SCM: Sunrise Clinical Manager
